# Beyond the Shape of Things: Infants Can Be Taught to Generalize Nouns by Objects’ Functions

**DOI:** 10.1177/0956797621993107

**Published:** 2021-06-10

**Authors:** Cecilia Zuniga-Montanez, Sotaro Kita, Suzanne Aussems, Andrea Krott

**Affiliations:** 1 School of Psychology, University of Birmingham; 2 Department of Psychology, University of Warwick

**Keywords:** noun learning, function bias, shape bias, vocabulary development, second-order generalization, open data, open materials

## Abstract

Two-year-olds typically extend labels of novel objects by the objects’ shape (*shape bias*), whereas adults do so by the objects’ function. Is this because shape is conceptually easier to comprehend than function? To test whether the conceptual complexity of function prevents infants from developing a function bias, we trained twelve 17-month-olds (function-training group) to focus on objects’ functions when labeling the objects over a period of 7 weeks. Our training was similar to previously used methods in which 17-month-olds were successfully taught to focus on the shape of objects, resulting in a precocious shape bias. We exposed another 12 infants (control group) to the same objects over 7 weeks but without labeling the items or demonstrating their functions. Only the infants in the function-training group developed a function bias. Thus, the conceptual complexity of function was not a barrier for developing a function bias, which suggests that the shape bias emerges naturally because shape is perceptually more accessible than function.

Infants learn words rapidly, especially object names (Frank et al., 2017). Early vocabulary development has profound and lasting consequences. For instance, children’s early vocabulary size and language skills predict later academic success (Bleses et al., 2016; Morgan et al., 2015). It is therefore important to study word-learning strategies that promote rapid vocabulary growth in infancy.

## The Role of Shape and Function in Word Learning and Generalization

When learning and extending object labels, infants, children, and adults prioritize different object properties depending on the task and information available (e.g., Diesendruck et al., 2003; Graham et al., 1999; Namy & Clepper, 2010). Older children and adults generalize labels on the basis of an object’s shape and function but prioritize function over shape when function information is available (Gathercole & Whitfield, 2001; Graham et al., 1999; Mueller Gathercole et al., 1995). Function is important because it provides information about an object’s intended use (Diesendruck et al., 2003) and therefore about its category (Booth & Waxman, 2002a). The knowledge of function helps to classify objects into the right category. Because the shape and function of objects are often correlated, shape can also indicate the object category. However, shape can sometimes be misleading (e.g., slippers that look like rabbits). Nevertheless, infants and younger children typically generalize object labels by shape, a strategy called *shape bias* (Gentner, 1978; Horst & Twomey, 2013; Hupp, 2015; Kucker et al., 2019; Landau et al., 1998; Perry & Samuelson, 2011). For example, Graham and colleagues (1999) showed that 3- to 5-year-olds generalize object labels on the basis of shape similarities when shape is pitted against function. Two- and 3-year-olds show a function bias only when the object’s function is demonstrated and explained (Diesendruck et al., 2003) or when children are allowed to manipulate and interact with the objects themselves (Kemler Nelson et al., 2000). Thus, whereas older children and adults prioritize function for generalizing object labels, infants and younger children prioritize shape.

## Why Do Infants Spontaneously Develop a Shape Bias but Not a Function Bias?

If adults name objects by both shape and function while prioritizing function (e.g., Graham et al., 1999), then why do infants initially develop a shape bias? There are two possible reasons. First, shape is, perceptually, a more easily accessible property than function. Shape can be identified immediately when one encounters an object (Graham & Poulin-Dubois, 1999) and is usually stable over time (Gentner, 1982), whereas function becomes apparent only after one manipulates an object and is usually transient (Landau et al., 1998). Second, shape is conceptually easier to comprehend than function (Gentner, 1978). Shape is a simple object property because it does not consist of qualitatively different subcomponents (a complex shape can be seen as a combination of simple shapes, but these components are again shapes). Function, however, is a complex object property involving causal relations among qualitatively different subcomponents (e.g., an agent performing the action, a function being performed, an object used to perform the action, and even a second object that the function is being performed on; Gentner, 1978; Gentner & Boroditsky, 2001). In addition, an object’s shape usually does not change over time, whereas an object’s function requires integration of information over time (e.g., a spoon is initially empty and then gets filled with food; Deák et al., 2002). Finally, shape is easier to individuate than function because shape has clear and stable boundaries (Gentner, 1982). Note that these reasons for the conceptual simplicity of shape have also been brought forward in the debate on why nouns (object names) are learned before verbs (action names; e.g., Gentner, 1982; Imai et al., 2008). Thus, the problem of mapping nouns to object functions resembles the problem of mapping verbs to actions.

If the preference of using shape over function in noun generalization is due to conceptual simplicity, then it should be difficult to train infants who are developing a shape bias to develop a function bias instead. It is possible to accelerate the emergence of a shape bias. Smith and colleagues (2002) conducted a 7-week training that highlighted the importance of shape for object labeling, and they found that 19-month-olds showed a precocious shape bias and that this training accelerated infants’ noun vocabulary growth outside the laboratory. If a similar training for function can enable 19-month-olds to use a function bias, then this would show that conceptual difficulty is not the obstacle that prevents a function bias from emerging spontaneously.

**Statement of Relevance**

Early language development is critical for children’s general development. It supports their ability to communicate, is essential for social interaction, and predicts their future academic performance. This study investigated how to promote word learning in 17-month-olds, who are at an age when children’s noun-learning strategies emerge. Research has suggested that a precocious word-learning bias based on easy-to-access perceptual features of objects (shapes) can be accelerated with training. We found that infants can also be taught a general word-learning strategy that requires a focus on conceptually more complex properties (functions). This finding is important for cognitive and developmental psychology, as it demonstrates infants’ cognitive abilities for learning the names of objects on the basis of their functions. It is also relevant for parents and early-years practitioners, as it could inform interventions for children who do not adopt typical word-learning strategies or are at risk of developing poor language skills.

## The Current Study

Do 2-year-olds spontaneously develop a shape bias, but not a function bias, because shape is conceptually easier to comprehend than function? If this is the case, then infants should not be cognitively ready to learn a function bias before their second birthday. To probe this question, we tested whether teaching infants to attend to function during object labeling leads to a function bias. We also investigated whether, like shape training, function training influences real-world vocabulary growth. We followed the same procedure as Smith and colleagues (2002), except that we taught infants to focus on function instead of shape. Thus, for 7 weeks, an experimenter taught 17-month-olds that the same nouns can be used to label objects with the same function. A control group was introduced to the same stimuli in a similar 7-week program but was not taught any labels or shown any functions. After training, participants completed a first-order generalization task (with familiar objects used in training) and a second-order generalization task (with novel objects not used in training) to test whether they would extend object labels on the basis of function, shape, or color. Furthermore, parents reported infants’ expressive vocabulary at the start and end of the study.

If infants can be taught to focus on function in word learning, then the function-training group should base their generalizations of familiar and novel object labels on function more often than the control group. If function training indeed leads to a function bias, then the function-training group, but not the control group, should extend labels by function more often than chance in both the first- and second-order generalization tasks.

If function training has an impact on word learning beyond the lab-based training, then it should accelerate the real-world vocabulary growth of the function-training group but not the control group. Because function training focuses on a strategy for noun learning, the function-training group should have a larger noun vocabulary than the control group at the end of the study than at the start. Given the similar challenge of mapping nouns to object functions and verbs to actions, teaching infants that objects with the same function (i.e., actions) have the same label might promote a general understanding that words can refer to actions. If this is the case, then the function-training group should have a larger verb vocabulary than the control group at the end of the study than at the start.

## Method

The raw data and materials of this study are available on OSF (https://osf.io/yra56/).

### Power analysis

Using G*Power (Version 3; Faul et al., 2007), we conducted two power analyses to determine our sample size. Our first power analysis was based on the study by Ware and Booth (2010). We calculated the effect size of the proportion of correct responses in the first block of their second-order generalization task. The means and standard deviations (Group 1: *M* = 0.53, *SD* = 0.23; Group 2: *M* = 0.33, *SD* = 0.13) showed an effect size of 1.07 (Cohen’s *d*). Using an error probability of 0.05 and a power of .80, we estimated that a sample size of 24 infants was required to achieve a similar effect size.

Our second power analysis was based on the study by Smith and colleagues (2002). We calculated the estimated effect size of the difference between the groups in the number of nouns produced at the end of the study. This was an estimated calculation, as the results provided by Smith and colleagues (2002) did not specify all the information required. The estimated means and standard deviations of both groups produced an estimated effect size of 0.71 (Cohen’s *d*). We converted this effect size to Cohen’s *f*, following the formula suggested by Cohen (1988), because we were interested in the number of participants required for a repeated measures design rather than only the difference between two means. To achieve the effect size of 0.357 (Cohen’s *f*) with an error probability of 0.05 and a power of .80, we estimated that a sample size of 18 participants would be required.

### Participants

Infants were recruited from Birmingham and the surrounding areas through community groups and playgroups, as well as via the databases of the Infant and Child Lab at the University of Birmingham and the Warwick Research with Kids Group at the University of Warwick. Our final sample included 24 typically developing 17-month-old infants, each of whom was randomly assigned to one of two groups: the function-training group (four girls; mean age = 17 months, 11 days; range = 17 months, 1 day–17 months, 28 days) and the control group (seven girls; mean age = 17 months, 10 days; range = 17 months, 2 days–17 months, 27 days). The groups did not differ significantly in age (*p* = .811), gender (*p* = .219), or socioeconomic status (*p* = .064; for more details, see Table S1 in the Supplemental Material available online). During the first-order generalization task, participants in the function-training group were on average 19.33 months old (*SD* = 0.26), and participants in the control group were on average 19.28 months old (*SD* = 0.44). During the second-order generalization task, participants in the function-training group were on average 19.57 months old (*SD* = 0.28), and participants in the control group were on average 19.58 months old (*SD* = 0.40).

The two participant groups had similar expressive-vocabulary sizes at the start and end of the study, as measured via parent report using the UK Communicative Development Inventories (UK-CDI) Words and Gestures questionnaire (Alcock et al., 2017). A comparison with the UK-CDI norms showed that the vocabulary sizes of each group were also typical for British English infants. Participant groups did not differ significantly in expressive-vocabulary size at the start of the study (function-training group: *M* = 56.1 words, 61st percentile based on the UK-CDI norms, *SD* = 59.7, range = 3–206; control group: *M* = 44.5 words, 54th percentile based on the UK-CDI norms, *SD* = 49.7, range = 8–190), *t*(22) = 0.52, *p* = .611, 95% confidence interval (CI) for the mean difference = [–34.9, 58.1]. The groups also did not differ significantly in their expressive-vocabulary size at the end of the study (function-training group: *M* = 136.4 words, *SD* = 103.6, range = 6–331; control group: *M* = 103.6 words, *SD* = 71.4, range = 33–280), *t*(22) = 0.90, *p* = .376, 95% CI for the mean difference = [–42.5, 108.2]. The UK-CDI (Alcock et al., 2017) was normed up to 18 months and therefore has no norms for the age of our participants at the end of the study (19 months).

Two assessments at Week 1 ensured that the two groups of infants did not differ significantly in their general attention or in their ability to pick up function similarities of objects (see Initial Assessments in the Supplemental Material). Six additional infants were excluded from the analysis because they either did not complete the study (five infants) or were exposed to an additional language at home (one infant). The remaining participants were from monolingual English-speaking homes and did not have any history of language delay or hearing problems.

The study was approved by the University of Birmingham Ethical Committee, and informed written parental consent was obtained. Parents were reimbursed for their travel expenses, and infants received a sticker during each lab visit as well as a book and a “Junior Scientist” diploma at the end of the final visit.

#### Socioeconomic-status calculation

The socioeconomic-status variable was calculated as a mean score of parent education, parent occupation, and household income. In one case, household income was not reported, so socioeconomic status was based on the remaining two scores.

#### Parent education

A 4-point scale was used to determine parent education (1 = *no formal education*, 2 = *less than an undergraduate/bachelor degree*, 3 = *undergraduate/bachelor degree*, 4 = *postgraduate education*). The average education score of both parents was calculated and then converted to a value between 0 and 1.

#### Parent occupation

Occupation of all participants was classified using the nine levels of the Office for National Statistics Standard Occupational Classification Hierarchy (Office for National Statistics, 2010). A score from 1 to 9 was assigned, with 9 being the highest value and 1 the lowest. The average score of both parents was calculated, apart from families with a stay-at-home parent, for which the occupation score was based only on the person who worked outside the home. This score was then converted to a value between 0 and 1.

#### Household income

Income was measured on a 4-point scale (1 = *less than £14,000*, 2 = *£14,001–£24,000*, 3 = *£24,001–£42,000*, 4 = *more than £42,000*). This score was then converted to a value between 0 and 1.

### Procedure

All participants were individually trained and assessed at the Infant and Child Lab at the University of Birmingham. The study took place over nine weekly visits: initial assessments (Week 1), training sessions (Weeks 1 to 7), and final assessments (Weeks 8 and 9). The same initial and final assessments were used for both participant groups, but the training differed.

#### Initial assessments

At Week 1, parents of all infants filled in the UK-CDI Words and Gestures questionnaire (Alcock et al., 2017), which was used to measure expressive-vocabulary size at the start of the study. A socioeconomic and general-development questionnaire was used to gather information about the infant’s general development, the infant’s family, and their socioeconomic status. It also informed the eligibility criteria for the study (e.g., no history of a language delay and English as the only language used at home). Additionally, two assessments at Week 1 (a sorting task and an attention task) ensured that the two groups of infants did not differ in their ability to pick up function similarities of objects or in their general attention (see Initial Assessments in the Supplemental Material for more information).

#### Training

Each participant was randomly assigned to either the function-training group or the control group.

##### Function-training group

**Fig. 1.**
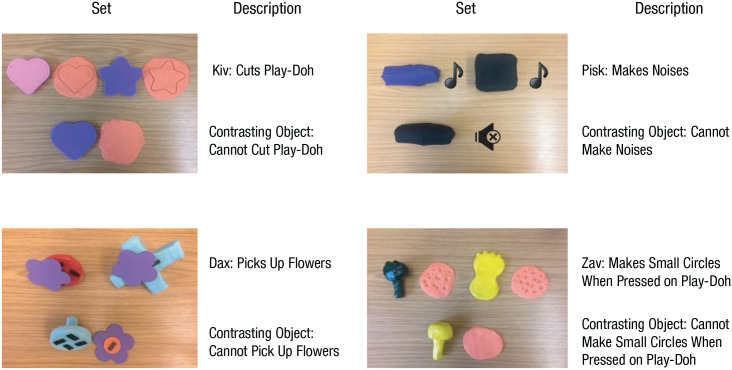
Sets of stimulus objects used for the function-training group and control group. Objects’ names were mentioned and their functions were explained and demonstrated only for the function-training group. Measurements and general descriptions of the stimulus objects can be found at https://osf.io/yra56/.

Infants in the function-training group were taught four novel words (*kiv*, *pisk*, *dax*, *zav*). Each word was introduced with a set of three novel objects: two referent exemplars that shared the same name and one contrasting object that did not share the name. The two referent exemplars also shared the same function with each other but differed in both color and shape. The contrasting object did not share the same function as the referent exemplar, but shared the same color as one of the exemplars and the same shape as the other exemplar (see Fig. 1). All objects were made from materials such as clay, cloth, or plastic, and each set of exemplars performed different functions. Kivs were used to cut Play-Doh, daxes were used to pick up flowers with magnets, pisks made noises when shaken, and zavs were used to make a pattern on Play-Doh when it was pressed. Note that object functions were not strongly correlated with object shapes.

Infants were presented with each set in a playlike manner for 3 min each (total time of 12 min), and the presentation order of all four object sets was randomized across participants. The experimenter first presented one exemplar while saying, for example, “Look, it’s a kiv and can cut Play-Doh.” Then the second exemplar was presented with a similar sentence (e.g., “Look, this is also a kiv and can cut Play-Doh”). The experimenter also demonstrated each function while explaining it. Halfway through the presentation of each set (after about 1.5 min), the contrasting object was presented. The experimenter tried to perform the same function as with the two exemplars and said, “Oh, no, this is not a kiv because it cannot cut Play-Doh.” The contrasting object was then taken away, and the experimenter and participant continued playing with the two exemplars. The same procedure was followed with the other three sets of objects. All object names were mentioned and all functions were described and performed at least 10 times per play session.

The same introduction and interaction with the same novel objects were repeated for six further weekly training sessions (Weeks 2 to 7), but the presentation order of object sets was randomized across participants and sessions. Nonfunctional play (e.g., hiding an object and finding it) occurred in some training sessions, especially in the last training session, to maintain infants’ interest.

##### Control group

During seven weekly sessions (Weeks 1 to 7), infants in the control group played freely with the same stimulus objects used in the function-training group. Any additional material (e.g., Play-Doh) required to demonstrate the object’s function was also included (see Fig. 1). Object names and functions were not mentioned or demonstrated. As in the function-training group, each play session lasted 12 min. In the later weeks, nonfunctional play was used to maintain infants’ interest.

#### Final assessments

During Weeks 8 and 9, infants from both groups were given the same final assessments (first-order and second-order generalization tasks). At the final visit, parents again completed the UK-CDI Words and Gestures questionnaire (Alcock et al., 2017) so we could measure expressive vocabulary at the end of the study.

##### First-order generalization task

In Week 8, all participants were presented with a first-order generalization task. This task consisted of two practice trials (practice phase) and eight test trials (test phase). Both groups were presented with exactly the same objects and materials, and the procedure of this test was identical for both groups.

In the practice phase, infants were presented with two practice trials to familiarize them with the procedure of the task. In each practice trial, a standard object (a long blue spoon) of a familiar category (spoons) was presented, accompanied by three objects sharing one property each with the standard object (function: a short orange spoon; color: a blue box; shape: a long brown block with a similar shape to the standard object’s shape). The experimenter said, “Look, this is a spoon and can be used to scoop food. Can you give me the other spoon?” In a second practice trial, another set of familiar objects with a different function was introduced (a blue ball as an exemplar, a round rattle, a blue dinosaur, and a green textured ball with oval bumps that made it look different from the exemplar), and the same procedure was followed as in the first practice trial. In order to move on to the test phase, infants had to correctly choose both target objects (the short orange spoon and the green ball). Both practice trials were repeated as necessary until infants responded correctly to both objects. Most infants chose the target objects during their first attempt. Infants who did not were shown the correct choice and responded correctly in their second attempt. One infant from the control group and two infants from the function-training group required two attempts to respond correctly.

**Fig. 2.**
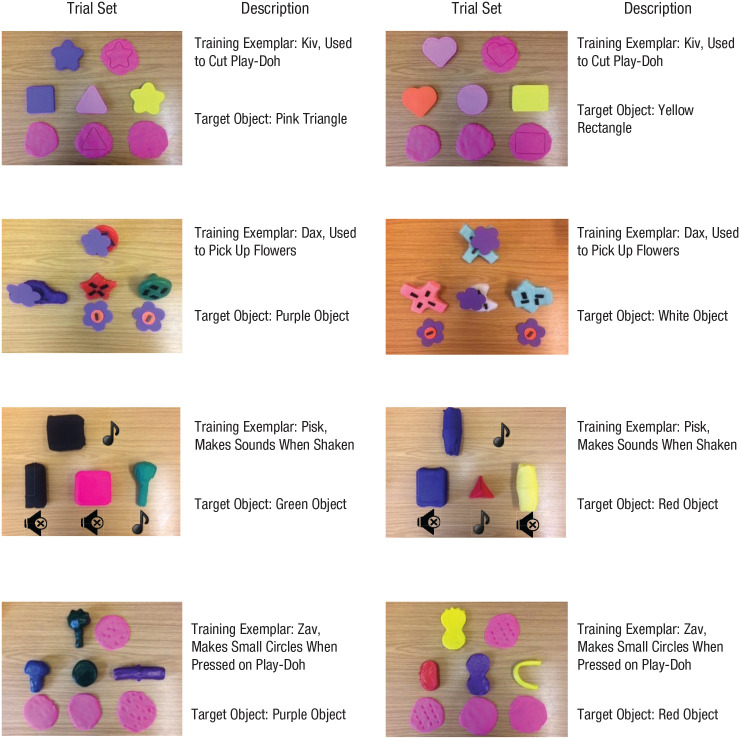
Sets of stimulus objects used during the first-order generalization task (Week 8). Each set consisted of one referent object (used during training) and three additional objects. One of the additional objects matched the standard object’s function, one matched its shape, and one matched its color. Measurements and general descriptions of the stimulus objects can be found at https://osf.io/yra56/.

The test phase consisted of eight trials—one trial for each exemplar used during the training weeks. In each trial, participants were shown one of the training exemplars and were asked to get an object that was referred to by the same name from a set of three possible options (see Fig. 2). Each of the three objects they could choose from shared only one property with the training exemplar (shape, color, or function). For each test trial, the experimenter named the familiar training exemplar, explaining and demonstrating the function in the same way as during the function-training sessions. For instance, she said, “This is a kiv and can be used to cut Play-Doh” while demonstrating the function of the kiv. She then said, “Now look at these ones.” After demonstrating in silence whether the three objects to choose from could perform the function of the familiar exemplar, she then asked, “Can you get the other kiv?” The eight trials were presented in one of two orders, counterbalanced across participants.

##### Second-order generalization task

In Week 9, all participants were presented with a second-order generalization task that consisted of a practice phase and a test phase. Both groups were shown exactly the same objects and materials, and the procedure was identical for both groups.

**Fig. 3.**
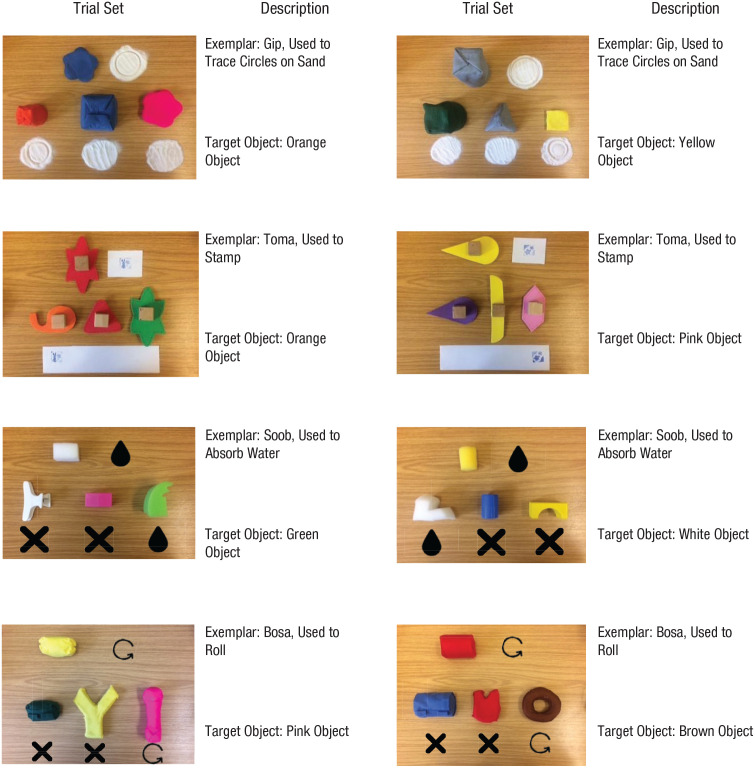
Sets of stimulus objects used during the second-order generalization task (Week 9). Each set consisted of one referent object and three additional objects. One of the additional objects matched the standard object’s function, one matched its shape, and one matched its color. None of the objects, labels, or functions had been used in the study before. Measurements and general descriptions of the stimulus objects can be found at https://osf.io/yra56/.

The practice phase was identical to that of the first-order generalization task in Week 8. In the test phase, participants were shown eight sets of completely new and unfamiliar objects, paired with four novel words and functions that participants had not encountered in the previous weeks (see Fig. 3). The same procedure as for the first-order generalization task was followed.

### Design and data analysis

#### First- and second-order generalization tasks

For both the first- and second-order generalization tasks, we calculated for each child the percentage of function choices out of their total number of choices. A choice was counted as a function choice if the chosen object shared the same function as the referent object. The total number of choices differed across participants because some choices were invalid. For both the first-order and second-order generalization tasks, the maximum number of choices was eight. However, in the first-order generalization task, three of our 24 participants had a total number of seven because on one trial they chose more than one object. For the second-order generalization task, five participants had a total of seven choices: Two participants chose more than one object for one trial, and three participants did not choose any object for one trial.

We compared the two groups’ percentage of function choices (function-training group vs. control group) in both the first-order and second-order generalization tasks using independent-samples *t* tests. We further compared each group’s percentage of function choices with chance (one out of three objects = 33.33%) using one-sample *t* tests.

#### Vocabulary growth

We analyzed the expressive vocabulary of the infants, as reported by their parents, using the UK-CDI Words and Gestures questionnaire (Alcock et al., 2017). We investigated vocabulary growth with a 2 (group: function training vs. control) × 2 (time: start of study vs. end of study) × 2 (word type: nouns vs. verbs) analysis of variance (ANOVA). Group was a between-subjects variable, and time and word type were within-subjects variables. The dependent variable was the total number of words infants produced. Two word categories were analyzed: nouns and verbs. For nouns, words in the following categories of the UK-CDI (Alcock et al., 2017) were included: animal words, vehicle words, words for toys, food and drink words, words for body parts, words for clothes, words for small household items, words for people, furniture words (17 items), and outside words (19 items). For verbs, all words from the category “action words” were included.

## Results

### First-order generalization

**Fig. 4.**
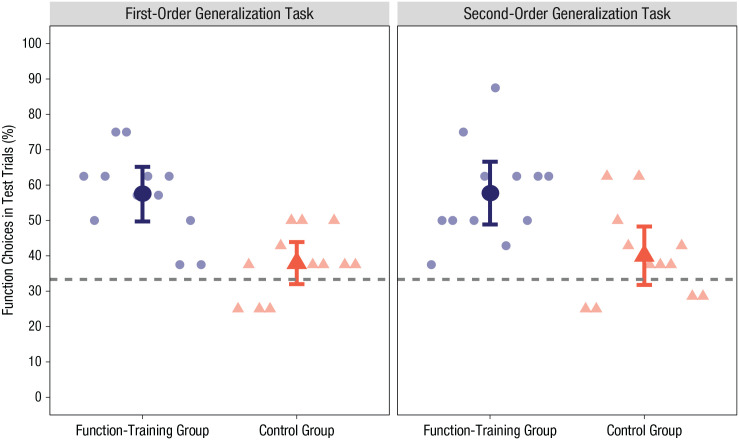
Percentage of function choices made by each group in the first-order and second-order generalization tasks. Large circles and triangles indicate group means. Error bars represent 95% confidence intervals. Small circles and triangles indicate individual participants’ data points. The dashed lines represent chance level (33.33%).

The left side of Figure 4 shows the average percentage of function choices for each group in the first-order generalization task. Infants in the function-training group (*M* = 57.44%, *SD* = 12.15) generalized object labels on the basis of function more often than did infants in the control group (*M* = 37.94%, *SD* = 9.36), *t*(22) = 4.40, *p* < .001, *d* = 1.79, 95% CI for the mean difference = [10.39, 28.67]. The function-training group also extended object labels on the basis of function significantly more often than chance (33.33%), *t*(11) = 6.87, *p* < .001, 95% CI for the mean difference = [16.38, 31.83], whereas the control group’s performance did not significantly differ from chance, *t*(11) = 1.70, *p* = .116, 95% CI for the mean difference = [–1.33, 10.56]. A stacked bar chart showing the percentages of all three choices in the first-order generalization task can be found in the Supplemental Material (Fig. S4).

### Second-order generalization

The right side of Figure 4 shows the average percentage of function choices for each group in the second-order generalization task. Infants in the function-training group (*M* = 57.73%, *SD* = 13.96) generalized object labels on the basis of function more often than infants in the control group (*M* = 40.03%, *SD* = 12.99), *t*(22) = 3.21, *p* = .004, *d* = 1.31, 95% CI for the mean difference = [6.28, 29.13]. The function-training group also extended novel labels by function significantly more often than chance (33.33%), *t*(11) = 6.05, *p* < .001, 95% CI for the mean difference = [15.53, 33.28], whereas the control group’s performance did not significantly differ from chance, *t*(11) = 1.78, *p* = .102, 95% CI for the mean difference = [–1.55, 14.95]. A stacked bar chart showing the percentages of all three choices in the second-order generalization task can be found in the Supplemental Material (Fig. S4).

### Vocabulary growth

**Fig. 5.**
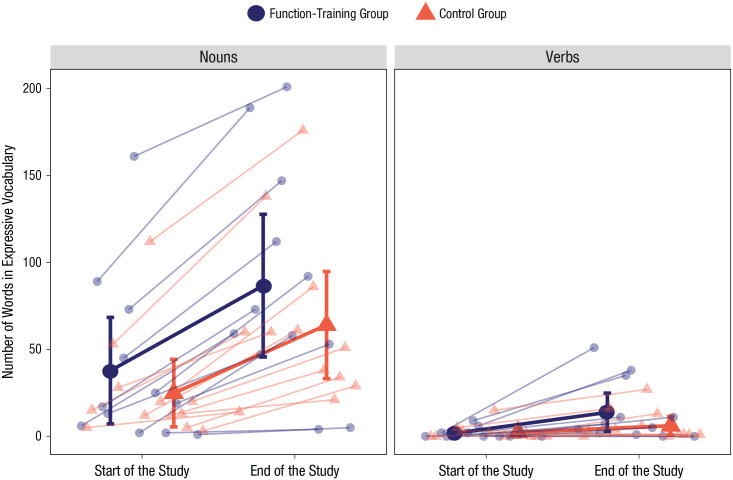
Number of nouns and verbs in the expressive vocabulary of the function-training group and the control group at the start of the study and at the end of the study. Large circles and triangles indicate group means. Error bars represent 95% confidence intervals. Small circles and triangles indicate individual participants’ data points. Connecting lines link performance of one individual or group across the two time points.

Figure 5 shows the average expressive noun and verb vocabulary sizes of both groups at the start and end of the study, as measured via parent report using the UK-CDI Words and Gestures questionnaire (Alcock et al., 2017). There was a significant main effect of time, *F*(1, 22) = 54.80, *p* < .001, η_*p*_^2^ = .71, and word type, *F*(1, 22) = 33.22, *p* < .001, η_*p*_^2^ = .60, on infants’ expressive vocabulary, but there was no significant main effect of group, *F*(1, 22) = 0.89, *p* = .356, η_*p*_^2^ = .03; interaction between time and group, *F*(1, 22) = 1.64, *p* = .213, η_*p*_^2^ = .07; or interaction between word type and group, *F*(1, 22) = 0.75, *p* = .395, η_*p*_^2^ = .03. However, there was a significant interaction between word type and time, *F*(1, 22) = 73.27, *p* < .001, η_*p*_^2^ = .76. That is, children’s noun vocabulary grew more than their verb vocabulary. Finally, there was no significant three-way interaction among group, time, and word type, *F*(1, 22) = 0.03, *p* = .861, η_*p*_^2^ = .01.

## General Discussion

This study has two key findings. First, infants in the function-training group generalized familiar (first-order generalization) and novel (second-order generalization) object labels on the basis of function more often than did infants in the control group. The function-training group did so more often than chance, whereas the control group did not. Thus, 17-month-olds can acquire a function bias as a successful word-learning strategy, which infants in the control group did not develop spontaneously. Second, the function-training group did not show accelerated real-world noun or verb vocabulary growth over the course of the study compared with the control group.

### Function training promotes first- and second-order noun generalization

The current study extends the word-learning literature in three important ways. First, our study is the first to show that infants can be taught a function bias for first-order noun generalization. Successful first-order generalization based on function had previously only been shown in 2- and 3-year-old children (Deák et al., 2002; Diesendruck et al., 2003; Kemler Nelson et al., 2000). Second, our study is the first to show an effect of function training on second-order generalization. Thus, it expands the existing literature on how to facilitate second-order generalization (Aussems & Kita, 2021; Perry et al., 2010; Samuelson, 2002; Smith et al., 2002; Ware & Booth, 2010). Third, and most importantly, our results show that 19-month-olds are cognitively ready to use function for word learning. Whereas Smith and colleagues (2002) accelerated a bias that infants would have developed naturally around the time of their training or soon thereafter, we introduced a bias that infants would not have developed until a few years later. This underlines that conceptual difficulty is not the obstacle that prevents infants from developing a spontaneous function bias.

Importantly, infants cannot be taught just any bias for word learning. Our training was likely successful because function is a relevant property for the naming and categorization of objects that infants encounter. Samuelson (2002) was not able to teach 15- to 20-month-olds a material bias using a training very similar to ours and that of Smith and colleagues (2002). Only a small number of objects that infants typically encounter are nonsolid objects that are organized and named by material (Samuelson, 2002). Therefore, infants appear to only pick up a bias that is strongly supported by their experience.

One limitation of this study is that we cannot know whether 7 weeks of training were necessary for infants to develop a function bias. Future studies should test infants’ generalization each week to assess how many training sessions are required.

### Why do infants initially develop a shape bias but not a function bias?

Our results suggest that the conceptual simplicity of shape is not the reason infants initially develop a shape bias instead of a function bias. Instead, infants seem to develop a shape bias because shape is, perceptually, a more easily accessible property than function. Shape can be identified as soon as infants encounter an object (Graham & Poulin-Dubois, 1999) and is stable over time (Gentner, 1982). In contrast, function requires manipulating an object and is mostly transient (Landau et al., 1998). Furthermore, many of the nouns that infants acquire refer to objects with correlated shapes and functions (e.g., spoon). Thus, infants can use the highly accessible cue—object shape—to eliminate erroneous referents for novel labels.

Our conclusion is orthogonal to the debate of how a shape bias emerges. Two accounts have been proposed in the literature. The first posits that a shape bias emerges through associative learning during noun learning (e.g., Landau et al., 1988; Smith et al., 2002). The second suggests that the focus on shape is a part of broader cognitive development, also seen in categorization behaviors (e.g., Bloom, 2000; Booth et al., 2005; Diesendruck et al., 2003) and in use of conceptual knowledge in noun extension (Booth & Waxman, 2002b). Neither of these accounts explains why shape is prioritized over function. Our answer to this question is compatible with both accounts.

### Why did function training not promote vocabulary growth?

Unlike the shape training by Smith and colleagues (2002), our function training did not promote vocabulary growth outside the laboratory above and beyond that of the control group. Interestingly, though, our control group showed a spontaneous preference for generalizing the familiar and novel labels by shape (see Fig. S4 in the Supplemental Material), whereas the control group of Smith and colleagues showed no bias whatsoever. For objects that infants typically interact with (e.g., spoons), either shape or function is often sufficient for the infants to know what they are called. Thus, our taught function bias might have been as beneficial for vocabulary growth as the spontaneous shape bias in our control group.

The above explanation suggests that function training may promote real-world vocabulary growth in populations that do not naturally develop a shape bias (e.g., children with autism spectrum disorder or late talkers; Field et al., 2016; Jones, 2003; Tek et al., 2008). This is an important topic for future research.

### Conclusion

To conclude, infants can be taught a function bias as a successful strategy for noun learning, and they can use this strategy even for novel words never encountered before (second-order generalization). Our study shows that by 19 months of age, infants can learn to systematically extend words on the basis of perceptually hard-to-access and conceptually complex information. Thus, it is unlikely to be the conceptual simplicity of shape, but rather its easy-to-access perceptual feature, that explains why the shape bias spontaneously emerges.

## Supplemental Material

sj-pdf-1-pss-10.1177_0956797621993107 – Supplemental material for Beyond the Shape of Things: Infants Can Be Taught to Generalize Nouns by Objects’ Functions Click here for additional data file. Supplemental material, sj-pdf-1-pss-10.1177_0956797621993107 for Beyond the Shape of Things: Infants Can Be Taught to Generalize Nouns by Objects’ Functions by Cecilia Zuniga-Montanez, Sotaro Kita, Suzanne Aussems and Andrea Krott in Psychological Science

## Transparency

*Action Editor:* Vladimir Sloutsky

*Editor:* Patricia J. Bauer

*Author Contributions*

C. Zuniga-Montanez and A. Krott designed the study. C. Zuniga-Montanez and S. Aussems recruited participants. Testing and data collection were performed by C. Zuniga-Montanez, who also analyzed the data under the supervision of A. Krott. All the authors interpreted the results and wrote the manuscript, and all the authors approved the final version for submission.

**Open Practices:** Raw data and analysis scripts, as well as the code scripts for figures and videos demonstrating the stimulus objects and their functions, have been made publicly available via OSF and can be accessed at https://osf.io/yra56/. The design and analysis plans for the study were not preregistered. This article has received the badges for Open Data and Open Materials. More information about the Open Practices badges can be found at http://www.psychologicalscience.org/publications/badges.

## References

[bibr1-0956797621993107] Alcock K. J. Meints K. Rowland C. F. (2017). UK-CDI words and gestures—Preliminary norms and manual. http://lucid.ac.uk/ukcdi

[bibr2-0956797621993107] Aussems S. Kita S. (2021). Seeing iconic gesture promotes first- and second-order verb generalization in preschoolers. Child Development, 92(1), 124–141. 10.1111/cdev.13392 32666515

[bibr3-0956797621993107] Bleses D. Makransky G. Dale P. Højen A. Ari B. A. (2016). Early productive vocabulary predicts academic achievement 10 years later. Applied Psycholinguistics, 37(6), 1461–1476. 10.1017/S0142716416000060

[bibr4-0956797621993107] Bloom P. (2000). How children learn the meanings of words. MIT Press. 10.1017/s0140525x01000139 12412326

[bibr5-0956797621993107] Booth A. E. Waxman S. R. (2002a). Object names and object functions serve as cues to categories for infants. Developmental Psychology, 38(6), 948–957. 10.1037/0012-1649.38.6.948 12428706

[bibr6-0956797621993107] Booth A. E. Waxman S. R. (2002b). Word learning is “smart”: Evidence that conceptual information affects preschoolers’ extension of novel words. Cognition, 84(1), 11–22. 10.1016/S0010-0277(02)00015-X 12062150

[bibr7-0956797621993107] Booth A. E. Waxman S. R. Huang Y. T. (2005). Conceptual information permeates word learning in infancy. Developmental Psychology, 41(3), 491–505. 10.1037/0012-1649.41.3.491 15910157

[bibr8-0956797621993107] Cohen J. (1988). Statistical power analysis for the behavioral sciences (2nd ed.). Erlbaum.

[bibr9-0956797621993107] Deák G. O. Ray S. D. Pick A. D. (2002). Matching and naming objects by shape or function: Age and context effects in preschool children. Developmental Psychology, 38(4), 503–518. 10.1037/0012-1649.38.4.503 12090481

[bibr10-0956797621993107] Diesendruck G. Markson L. Bloom P. (2003). Children’s reliance on creator’s intent in extending names for artifacts. Psychological Science, 14(2), 164–168. 10.1111/1467-9280.t01-1-01436 12661679

[bibr11-0956797621993107] Faul F. Erdfelder E. Lang A.-G. Buchner A. (2007). G*Power 3: A flexible statistical power analysis program for the social, behavioral, and biomedical sciences. Behavior Research Methods, 39(2), 175–191. 10.3758/BF03193146 17695343

[bibr12-0956797621993107] Field C. Allen M. L. Lewis C. (2016). Attentional learning helps language acquisition take shape for atypically developing children, not just children with autism spectrum disorders. Journal of Autism and Developmental Disorders, 46(10), 3195–3206. 10.1007/s10803-015-2401-1 25733159

[bibr13-0956797621993107] Frank M. C. Braginsky M. Yurovsky D. Marchman V. A. (2017). Wordbank: An open repository for developmental vocabulary data. Journal of Child Language, 44(3), 677–694. 10.1017/S0305000916000209 27189114

[bibr14-0956797621993107] Gathercole V. C. M. Whitfield L. C. (2001). Function as a criterion for the extension of new words. Journal of Child Language, 28(1), 87–125. 10.1017/S030500090000458X 11258016

[bibr15-0956797621993107] Gentner D. (1978). What looks like a jiggy but acts like a zimbo?: A study of early word meaning using artificial objects. Papers and Reports on Child Language Development, 15. ERIC. https://eric.ed.gov/?id=ED196301

[bibr16-0956797621993107] Gentner D. (1982). Why nouns are learned before verbs: Linguistic relativity versus natural partitioning. In Kuczaj, II S. (Ed.), Language development: Vol. 2. Language, thought and culture (pp. 301–334). Erlbaum.

[bibr17-0956797621993107] Gentner D. Boroditsky L. (2001). Individuation, relativity, and early word learning. In Bowerman M. Levinson S. (Eds.), Language culture and cognition: Language acquisition and conceptual development (pp. 215–256). Cambridge University Press. 10.1017/CBO9780511620669.010

[bibr18-0956797621993107] Graham S. A. Poulin-Dubois D. (1999). Infants’ reliance on shape to generalize novel labels to animate and inanimate objects. Journal of Child Language, 26(2), 295–320. 10.1017/S0305000999003815 11706467

[bibr19-0956797621993107] Graham S. A. Williams L. D. Huber J. F. (1999). Preschoolers’ and adults’ reliance on object shape and object function for lexical extension. Journal of Experimental Child Psychology, 74(2), 128–151. 10.1006/jecp.1999.2514 10479398

[bibr20-0956797621993107] Horst J. S. Twomey K. E. (2013). It’s taking shape: Shared object features influence novel noun generalizations. Infant and Child Development, 22, 24–43. 10.1002/icd.1768

[bibr21-0956797621993107] Hupp J. M. (2015). Development of the shape bias during the second year. The Journal of Genetic Psychology, 176(2), 82–92. 10.1080/00221325.2015.1006563 25775081

[bibr22-0956797621993107] Imai M. Li L. Haryu E. Okada H. Hirsh-Pasek K. Golinkoff R. M. Shigematsu J. (2008). Novel noun and verb learning in Chinese-, English-, and Japanese-speaking children. Child Development, 79(4), 979–1000. 10.1111/j.1467-8624.2008.01171.x 18717902

[bibr23-0956797621993107] Jones S. S. (2003). Late talkers show no shape bias in a novel name extension task. Developmental Science, 6(5), 477–483. 10.1111/1467-7687.00304

[bibr24-0956797621993107] Kemler Nelson D. G. Russell R. Duke N. Jones K. (2000). Two-year-olds will name artifacts by their functions. Child Development, 71(5), 1271–1288. 10.1111/1467-8624.00228 11108096

[bibr25-0956797621993107] Kucker S. C. Samuelson L. K. Perry L. K. Yoshida H. Colunga E. Lorenz M. G. Smith L. B. (2019). Reproducibility and a unifying explanation: Lessons from the shape bias. Infant Behavior and Development, 54, 156–165. 10.1016/j.infbeh.2018.09.011 30343894 PMC6393169

[bibr26-0956797621993107] Landau B. Smith L. B. Jones S. S. (1988). The importance of shape in early lexical learning. Cognitive Development, 3(3), 299–321. 10.1016/0885-2014(88)90014-7

[bibr27-0956797621993107] Landau B. Smith L. Jones S. (1998). Object shape, object function, and object name. Journal of Memory and Language, 38(1), 1–27. 10.1006/jmla.1997.2533

[bibr28-0956797621993107] Morgan P. Farkas G. Hillemeier M. Scheffner Hammer C. (2015). 24-month-old children with larger oral vocabularies display greater academic and behavioral functioning at kindergarten entry. Child Development, 86(5), 1351–1370. 10.1111/cdev.12398 26283023 PMC4567967

[bibr29-0956797621993107] Mueller Gathercole V. C. Cramer L. J. Somerville S. C. Jansen op de Haar M. (1995). Ontological categories and function: Acquisition of new names. Cognitive Development, 10(2), 225–251. 10.1016/0885-2014(95)90010-1

[bibr30-0956797621993107] Namy L. L. Clepper L. E. (2010). The differing roles of comparison and contrast in children’s categorization. Journal of Experimental Child Psychology, 107(3), 291–305. 10.1016/j.jecp.2010.05.013 20609449

[bibr31-0956797621993107] Office for National Statistics. (2010). ONS Standard Occupational Classification (SOC) Hierarchy. https://onsdigital.github.io/dp-classification-tools/standard-occupational-classification/ONS_SOC_hierarchy_view.html

[bibr32-0956797621993107] Perry L. K. Samuelson L. K. (2011). The shape of the vocabulary predicts the shape of the bias. Frontiers in Psychology, 2, Article 345. 10.3389/fpsyg.2011.00345 PMC3222225 22125547

[bibr33-0956797621993107] Perry L. K. Samuelson L. K. Malloy L. M. Schiffer R. N. (2010). Learn locally, think globally: Exemplar variability supports higher-order generalization and word learning. Psychological Science, 21(12), 1894–1902. 10.1177/0956797610389189 21106892 PMC3144952

[bibr34-0956797621993107] Samuelson L. K. (2002). Statistical regularities in vocabulary guide language acquisition in connectionist models and 15-20-month-olds. Developmental Psychology, 38(6), 1016–1037. 10.1037/0012-1649.38.6.1016 12428712

[bibr35-0956797621993107] Smith L. B. Jones S. S. Landau B. Gershkoff-Stowe L. Samuelson L. (2002). Object name learning provides on-the-job training for attention. Psychological Science, 13(1), 13–19. 10.1111/1467-9280.00403 11892773

[bibr36-0956797621993107] Tek S. Jaffery G. Fein D. Naigles L. R. (2008). Do children with autism spectrum disorders show a shape bias in word learning? Autism Research, 1(4), 208–222. 10.1002/aur.38 19360671 PMC2630708

[bibr37-0956797621993107] Ware E. A. Booth A. E. (2010). Form follows function: Learning about function helps children learn about shape. Cognitive Development, 25(2), 124–137. 10.1016/j.cogdev.2009.10.003 PMC2879495 20526449

