# Supplementary material for: Beyond the Shape of Things: Infants Can Be Taught to Generalize Nouns by Objects’ Functions
Source: Psychol Sci. 2021 Jun 10;32(7):1073–85. doi: 10.1177/0956797621993107 (PMC8641134; doi:10.1177/0956797621993107)
Supplement: sj-pdf-1-pss-10.1177_0956797621993107 – Supplemental material for Beyond the Shape of Things: Infants Can Be Taught to Generalize Nouns by Objects’ Functions [file sj-pdf-1-pss-10.1177_0956797621993107.pdf]

Supplemental Material

Table S1

Demographics.

	Function-training group			Control group		
	n	*M*	*SD*	*n*	*M*	*SD*
Age at start of the study (months)		17.39	0.31		17.36	0.26
Age at week 8 (months)		19.33	0.28		19.28	0.44
Age at week 9 (months)		19.57	0.29		19.58	0.40
Gender						
Male	8			5		
Female	4			7		
Age of first word (months)		11.28[a]	2.42		11.62[a]	3.33
SES score		0.82	0.12		0.69	0.19

a. No information for 5 participants in the function-training group and 4 in the control group.

b. The raw data and analysis scripts are available via the Open Science Framework via https://osf.io/yra56/?view_only=9ae3702551ca4d51a10d69dea5e74dff

Initial Assessments

To rule out that the two groups differed in their ability to pick up function similarities or in their attention to actions, a sorting and attention task were administered at the start of the study.

Sorting task

Method. Infants were presented with two sets of eight objects (16 in total; four to demonstrate the task to the infants, 12 for the task the infants performed). Objects were red or green and had one of two novel shapes (see Figure S2). One set of objects was made of wax (could be used to draw) and one of clay (could not be used to draw).

Objects were mixed and placed on a table in front of the child, along with a paper sheet and two containers. The researcher said: "*We are going to play a game; we are going to put objects together that are the same"*. She then drew on the paper with one of the wax objects and said: *"Look, this one can be used to draw"*, and placed the object in one container while saying: *"This one goes here"*. She then picked up one of the clay objects that had the same shape and colour as the wax object, tried to draw with it and said: "*Oh no, this one cannot be used to draw"*, and placed it in the other container while saying: *"So this one goes here".* The researcher demonstrated how to sort two more objects and said: *"Now it is your turn, can you help me put the objects that are like these ones here (pointing to one container), and the objects that are like these ones here (pointing to the other container)?"*. Participants then sorted 12 objects.

Results. The function-training group sorted objects by function in 50.41% of the trials (SD = 7.30) and the control group in 46.54% (SD = 7.25). Performance did not differ significantly between groups, $t(22) = 1.30$, $p = .206$, 95% CI = [-2.28, 10.04], neither did it differ significantly from chance in either group (function-training group: $t(11) = 0.20$, $p = .847$, 95% CI = [-4.22, 5.05]; control group: $t(11) = -1.65$, $p = .127$, 95% CI = [-8.07, 1.15]). Thus, both groups were unable to sort objects by function at the start of the study and did not differ in this ability.

Figure S2. Sorting task stimuli.

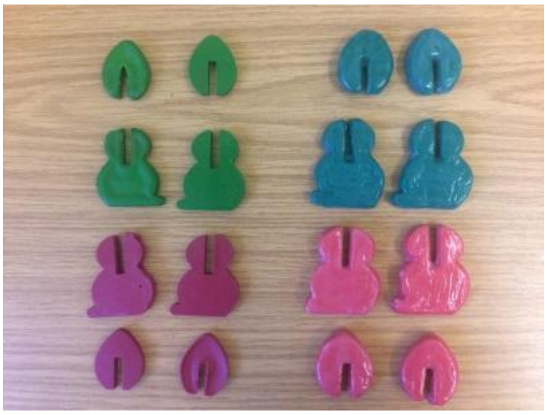

Note. One set of objects (left) was made of wax (could be used to draw) and one set (right) was made of clay (could not be used to draw). Measurements of the sorting task stimuli are available via https://osf.io/yra56/?view_only=9ae3702551ca4d51a10d69dea5e74dff

Attention Task

Method. Infants sat on their caregiver's lap, approximately 60 cm away from a monitor. An eye-tracker (EyeLink 1000 with 9-point calibration) recorded infants' looking times during five video clips. Each video lasted 35 seconds and showed a person performing a simple action with an unusual object on the right or left side of the screen. On the opposite side of the action, a static distracting object was visible (see Figure S3). For the first 10 seconds, no object was moved. For the following 25 seconds, the target object was moved and used to perform a function (e.g., a mango splitter was used to cut Play-Doh). Participants viewed five similar videos, which featured different objects and actions. The presentation order of the videos was randomised for each participant, and the side on which the function was performed was counterbalanced across participants. For the analysis, we defined three regions of interest (ROIs): action, distractor, and face.

Figure S3. Still frames of a video from the attention task

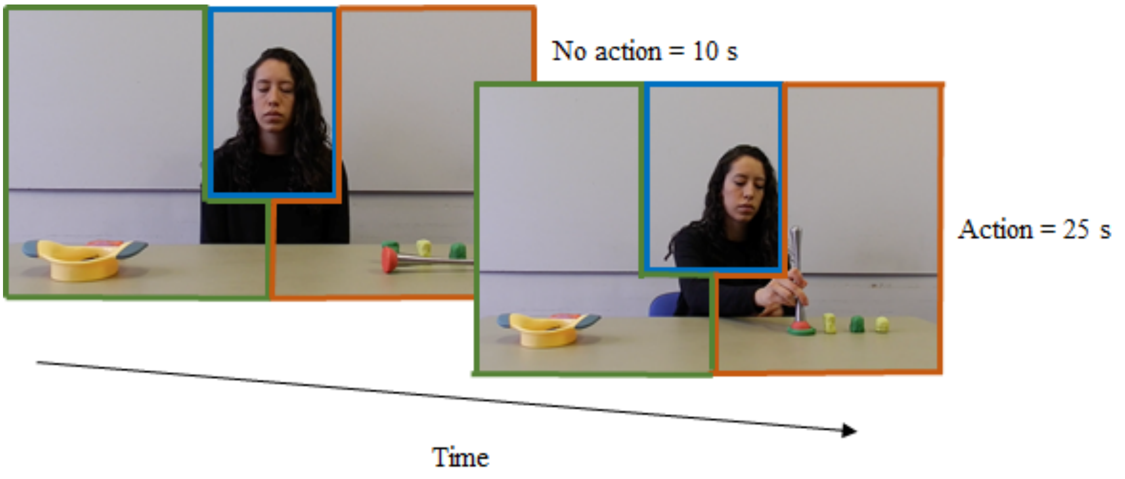

Note. Videos were divided into two time windows and three regions of interest (ROIs) (blue: face ROI, green: distractor ROI, orange: action ROI).

Results. To assess if infants' attention was equally focused on actions in both groups, the proportion of looking times towards the action ROI was calculated for each video before and during the action, using the following equation: mean fixation on action ROI / (mean fixation on action ROI + mean fixation on distractor ROI + mean fixation on face ROI). Proportions of looking times of the two groups were very similar. Both groups looked longer at the action ROI during the action than before (function-training group: M_{before} = 0.14, *SD* = 0.08, M_{during} = 0.66, *SD* = 0.23; control group: M_{before} = 0.15, *SD* = 0.10; M_{during} = 0.60, *SD* = 0.30). This was confirmed by a 2 (group: function-training vs. control) x 2 (time: before vs. during action) ANOVA, which showed a main effect of time, $F(1, 22) = 95.54$, $p < .001$, $\eta_p^2 = .81$, but no main effect of group, $F(1, 22) = 0.11$, $p = .744$, $\eta_p^2 = .00$, or an interaction between group and time, $F(1, 22) = 0.45$, $p = .508$, $\eta_p^2 = .02$. Thus, both groups focused their attention on actions in a similar way.

First- and Second-Order Generalisation

Figure S4.

Percentage of function, shape, and colour choices (*y*-axis) by group (*x*-axis) in first-order (left panel) and second-order (right panel) generalisation tests

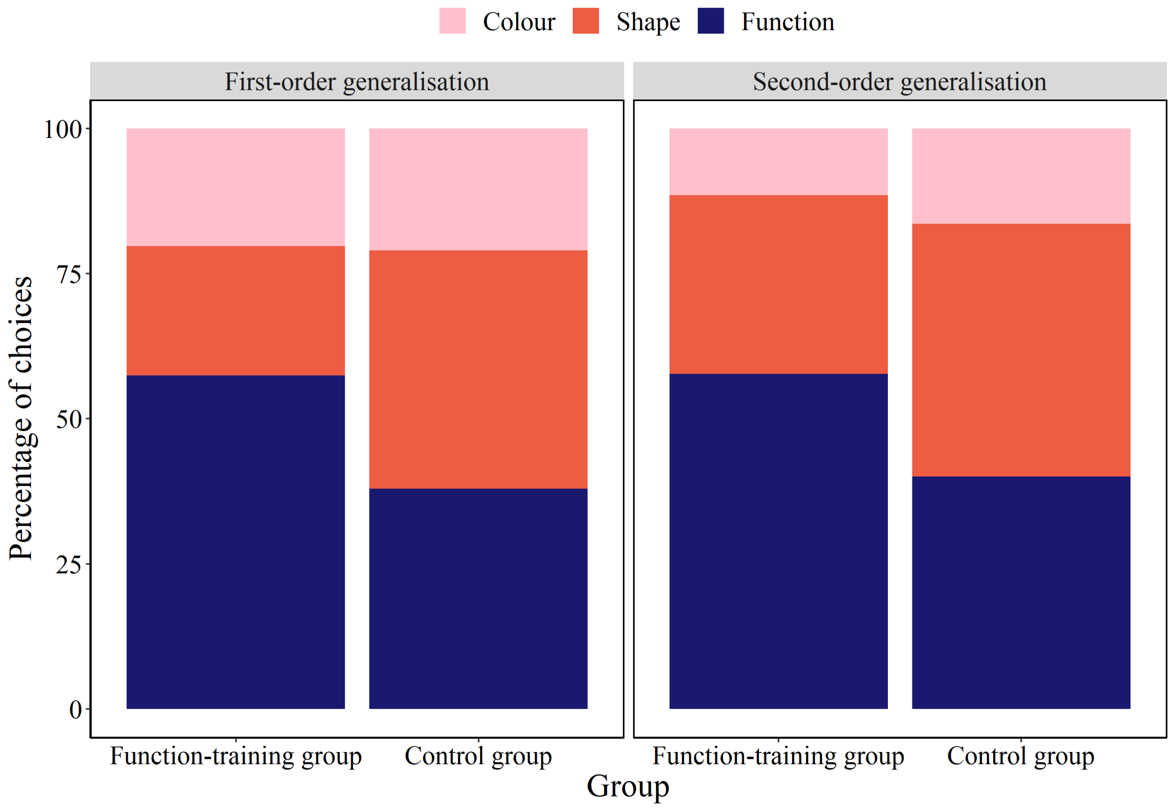

Note. The control group extended labels by shape significantly above chance (33.33%) in both the first-order (M = 41.07%, SD = 12.03), $t(11)$ = 2.22, p = .048, 95% CI = [0.92, 15.39]) and second-order generalisation test (M = 43.60%, SD = 12.59), $t(11)$ = 2.82, p = .017, 95% CI = [2.27, 18.27]). The control group thus showed a spontaneous shape bias.
